# Supplementary material for: D-amphetamine alters the dynamic ECoG activity distribution patterns in the rat neocortex
Source: Sci Rep. 2025 Nov 4;15:38457. doi: 10.1038/s41598-025-26688-5 (PMC12586689; doi:10.1038/s41598-025-26688-5)
Supplement: Supplementary file 1 — Supplementary Material 1 [file 41598_2025_26688_MOESM1_ESM.pdf]

D-amphetamine alters the dynamic ECoG activity distribution patterns in the rat neocortex

Astrid Mellbin[1 *], Henrik Jörntell[1], Fredrik Bengtsson[1]

[1]Neural Basis of Sensorimotor Control, Department of Experimental Medical Science, Biomedical Centre, Lund University, SE-223 62, Lund, Sweden

*Corresponding author

E-mail: Astrid.Mellbin@med.lu.se

Supplementary figure S1: Example of evoked response to stimulation

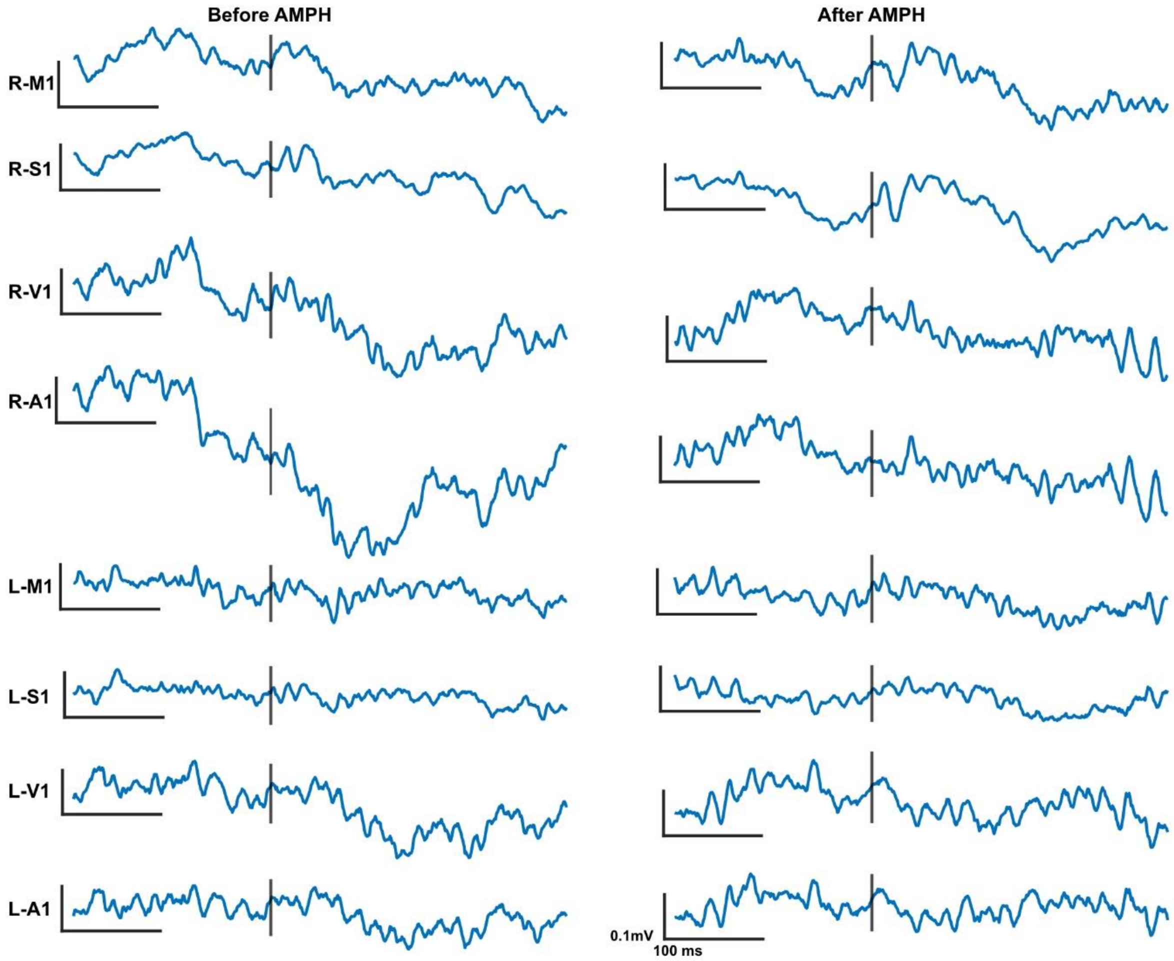

The average of the ECoG trace from 200 ms before to 300 ms after each stimulation impulse for the 0.3 Hz stimulation of the forepaw. The column on the left show the response before D-amphetamine administration and the column on the right show after. Each row represent an electrode, on either the right (R-) or the left (L-) hemisphere.

Supplementary figure S2: ECoG data plotted in the PC space in figure 3B

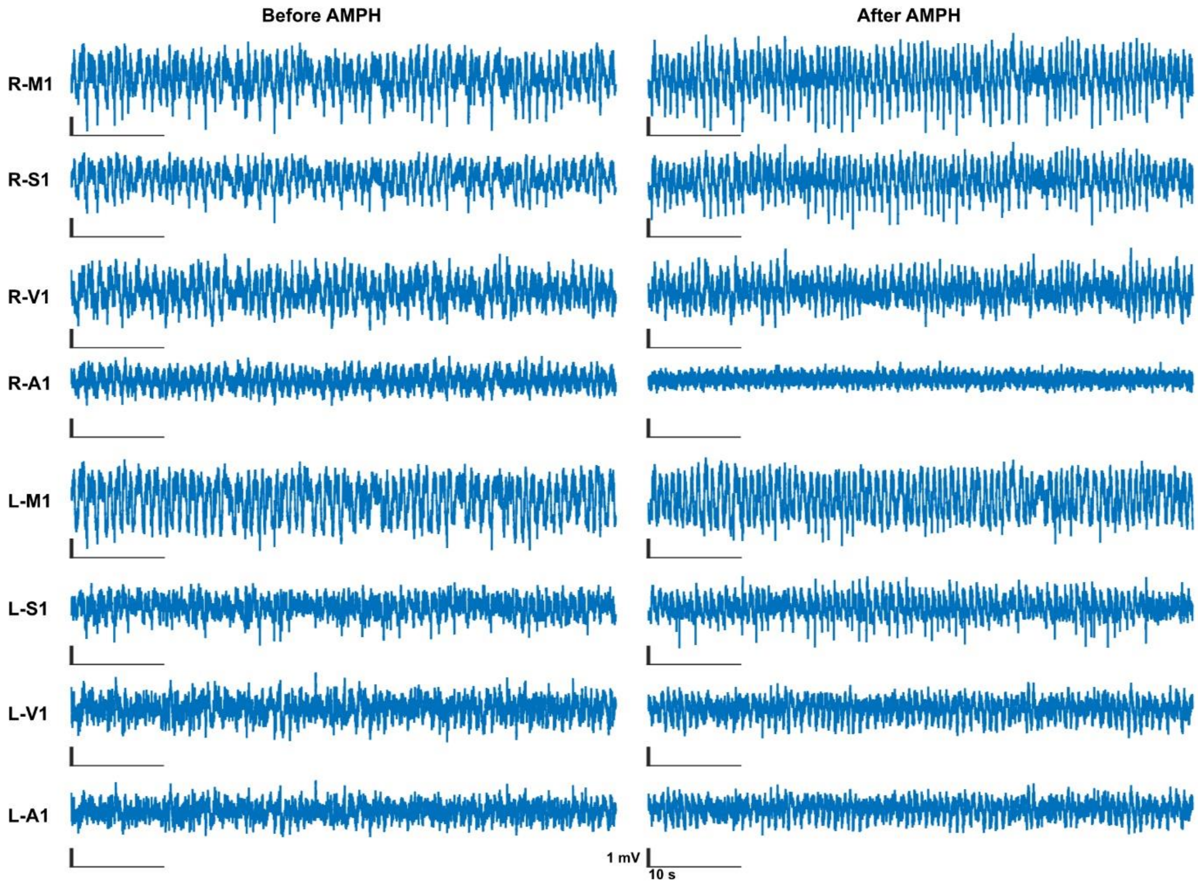

1 minute of raw ECoG traces that were plotted in the PC space in figure 3B. The left column show raw traces from before D-amphetamine and the column on the right show raw traces after. Rows correspond to each individual area recorded, either on the right (R-) or on the left (L-) hemisphere.
